# The Moralities of Medicine*An address by Dr. Charles M. Rosser, Dallas, Texas, retiring President Medical Association of the Southwest, Hot Springs meeting, October 10, 1907.

**Published:** 1907-11

**Authors:** 


### TEXAS MEDICAL JOURNAL.

Established July, 1885.

F. E. DANIEL, M. D., - - - - Editor, Publisher and Proprietor

PUBLISHED MONTHLY.—SUBSCRIPTION $1.00 A YEAR.

VOL. XXIII. AUSTIN, NOVEMBER, 1907. No. 5.

The publisher is not responsible for the views of contributors.

For the Texas Medical Journal.

### The Moralities of Medicine.*

*Mr. President, Ladies and Gentlemen:*

In the exercise of a brief authority, conferred, perhaps, by undue partiality one year ago, I have the honor to speak upon this occasion for the medical men of Missouri, Kansas, Arkansas, Oklahoma and Texas; but, complimentary as was your courtesy, important as is the opportunity, and appreciative as must be the distinction, I promise that in what I may say as your retiring first president, I shall endeavor to be neither technical nor tedious.

Men must ever meditate upon demonstrations of genius, and in this way they marvel not more because of discoveries and improvements thereto, than at their long delay, and now after a second successful session of this Medical Association of the Southwest, Topeka, St. Louis, Hot Springs, Dallas, Oklahoma and other thoughtful cities in the progressive States of our prescribed territory must give to Kansas City a creative credit in this connection, which merits historic record as we pass toward a fulfillment of the prophecies accompanying the original proposition to organize. It is due also to say that the primary thought was the product and projection of the well-reputed Academy of Medicine, which has long reflected credit upon Kansas City, and that the motive for the movement was endorsed by that body at an annual banquet and in the hearing of the then president of the American Medical Association, who personally gave his approval to the ideas as advanced. Visits were then made to the societies of the several States

*An address by Dr. Charles M. Rosser, Dallas, Texas, retiring President Medical Association of the Southwest, Hot Springs meeting, October 10, 1907.

of the Southwest, and committees secured by their appointment, which jointly inaugurated a provisional organization shortly afterwards. This only preceded a permanent establishment from which has developed the work of today. At the time now referred to a merger was made with the Southwestern Tri-State Society (Texas, Indian Territory and Oklahoma) upon terms of mutual satisfaction and agreement, the latter forming a tangible nucleus for immediate action, and I think it not inappropriate to remark upon the sentimental sacrifice involved in allowing the original organization to lose its individual identity.

This has been a year of interest and anxiety, as we have day by day approached a time, when, if at all, the existence of the Institution was to be justified and its future usefulness assured. Those conversant could hardly doubt either affirmation, since our purposes and plans have been in conformity to those of like patriotism, and upon lines approved by the test of human experience. Having no concern with matters of legislation, and broadly beyond any friction of locality, it occupies a position midway between the organizations of the States and Nations with those necessary burdens by giving time only to the social and the scientific. In as much as there is everywhere a harmony with the cherished principles maintained by the American Medical Association, and as there is no conflict in either creed or constitution, we shall assume a prerogative to proceed individually in the sense of determining details that are essential to our local conditions and have no reasonable doubt of a complete and proper recognition on the part of the national body when our committee shall have had opportunity to present our cause and claims.

This society has an especial mission, and it is imperative. To it is entrusted the duty of doing for the profession and people of our geographical relationship that which no other agency can perform, viz., the dissemination of medical knowledge, and that of the most definite and modern character, until all that is of value to any of us shall be available to each of us whether located in the crowded city or upon the crossroads of our common country.

The American Medical Association, meeting as it does ordinarily in the North and East, is only accessible to those of our remote, though vast and populous territory, after the expenditure of much time and specific expense, and its splendid program attracting to its sections so many thousand men, it is impossible to only a small minority of those who go to have part in its proceedings, and to become working factors in its progress.

A miniature American Medical Association in our midst may for us accomplish in the South and West what has been observed in the North and East, and, while it will be well for all who may to make the sacrifice required for attendance upon the national gatherings, yet in any and all events this society, whether recognized as an auxiliary or left alone to its own direction, will live and grow as it works out its splendid destiny. Its ideal result will be reached when the medical men of the Southwest shall be able as they should to meet the duties growing out of our professional responsibilities,—responsibilities which attach to citizenship and those higher obligations which are imposed by the delicate and delightful relations which the doctor is permitted to sustain toward his fellowman. In his preparation for these, the study and the practice of medicine presents superior forces for enhancement of natural endowments and stimulation of philosophic sentiments suitable to a medical atmosphere.

Contrary to a popular notion and to interpretations by mediocre or meaner men, the ethics of the profession is neither ridiculous nor restrictive, the efforts of mediocre or meaner men to so interpret notwithstanding.

Is it mechanics that are of interest? Study the anatomy of man, the acknowledged masterpiece, and his physiology, which exhibits more minutely than any other organism in activity a delicate dependency of component parts.

Would we volunteer in defense of the public health against a myriad hidden foes? Consult the microscope for their discovery and isolation and by laboratory methods learn their plans of propagation and habits of attack.

Are we speculative or philosophic? Trace by faithful finger the pathological process from first cause of disorder to termination of disease, having along the way an exercise for all capacities whether for prophecy in judgment or courageous skill in meeting conditions.

In giving to the mother the smile of her firstborn, or bending over the couch of death when mortal man gives up the struggle, the entire gamut of sentiment may play upon the human heartstrings in harmonies attuned to hope's bright hour, or subdued by sympathy to the silence of despair.

As a matter of mutual congratulation and concern, I shall affirm that a dictum of social economy which requires "Much in return when much is given" lays tribute upon the doctor for a higher order of intellectual conception, of adaptability and civic usefulness, than in due ratio should be taxed against individuals of any other class. The apparent arrogance of this assumption disappears after a review of the distinctive principles which separately underlie the several comparable callings in which men of similar temperament and kindred primary training may agreeably engage.

In this connection we may mention academic instruction, business, legal pursuits, and that diviner science which relates to a Christian life of faith and future.

Each of these are ennobling in their nature and indispensable to the welfare of the world, and yet educational systems are evolved and understood; commerce is conducted upon rather accurately adjusted rules applicable to its varied complexities; the law, statutory and of recent record, interpreted by courts and decided by juries can not be a mental gymnasium, and theology is amply founded upon inspiration and revelation, the verities of which are proclaimed in a universe of things about us and by the inner consciousness of all God's creatures so endowed.

All these operating as they do under conditions with more or less fixed fundamentals and of appropriate dogmatism in most matters of doctrine, students need not enter that labyrinthian search for truth to which the devotee to medical science is apprenticed for a life work in advance.

Recognizing no authorities other than individual judgment, and clinging to no criterion, it rewrites its ritual upon the page of last experience, a method to meet every menace to the body constitution influenced by the varying latitudes, the yearly seasons, and even the changing winds.

A mind so profoundly employed should early abdicate, or, persevering, measure to a mental grasp when finding its purposes imperative, can presuppose no impossibility and will regard no result as miraculous, although the thought of it rested wholly in desire.

The medical profession is unique in other respects. In holding as a first duty the prevention of disease, it unselfishly seeks to render its offices for which pecuniary charge is made less often necessary and, therefore, its graver duties of not so much demand.

In its written code against the employment of secret remedies it declares for the freest distribution of ideas and discoveries, whether in materia medica or methods, thus denying to its individual members the peculiar financial benefits which ordinarily obtain for inventors and ingenious men engaged in other lines of labor.

Charity has from its hand more service of that kind which involves personal sacrifice, individual inconvenience with incident pecuniary loss than it claims from all other sources combined, and, without respect to creed or color, situation or condition, humanity at large must ever look to those legitimately engaged in the healing art for a medical and surgical advice and service for which there can be no substitute.

The profession stands singly again in the light of the fact that it is the only institution or agency which gives practical assent to the sacredness of human life.

Does commerce, compelling as it does its appalling hazard to artisan and operator, recognize the sacredness of human life? If so, why the almost prohibitory rates required by insurance companies against disability and death on account of the unsafe occupations of those who extend civilized conditions by exploration, who construct or are brought in contact with undisputed elements of danger to limb and life as a part of their employment?

The law does not recognize the sacredness of human life and may not in respect to public policy, also legislators would repeal enactments providing for capital punishment, even in cases of capital crime.

Governments, while conscripting its citizens for military duty or maintaining standing armies upon land and sea at a beggar's price, do not recognize the sacredness of human life; but, on the contrary, propose it as a policy that for political injury or even diplomatic infraction, the result final shall be a test of man's ability to destroy his fellowman, an awful arbitrament which can only be abolished through a universal and, I fear an impossible, international peace.

The scriptural authority which gave as an award "an eye for an eye and a tooth for a tooth," and declares that "whoso sheddeth man's blood by man shall his blood be shed," denies to a religion, the most forceful of all factors for successful civilization, its belief in the *absolute* sacredness of human life, thus leaving to the science and art of medicine alone the supremest human championship known from creation to this good hour.

For whether as in morgue and laboratory the basis of the science is given its firmer foundation, in the hut of destitution, in the mansions where live the men of millions, or in hospitals and sanitaria, the call is for advice and skill, professional endeavor is ever the same,—to prevent disease, to lessen suffering and to stay man's entrance toward eternity! And the glory of the profession is not so much in its men of brilliant mark as in the character and qualifications of the rank and file, not more in the advanced learning of its leaders than in the promptness and uniformity with which scientific discoveries and clinical observations become common property for the service of the suffering sick wherever found.

With a knowledge of responsibilities of such serious import, and possibilities so far-reaching and obligatory, the profession stands with uncovered head, but with heart that is proudly brave. Humbled in the presence of its stupendous task, yet standing in the light for the good of all it may gain and give, it leads no clandestine life, but meets its duties daily with unfaltering faithfulness—a universal friend.

In a notable address by Lord Salisbury he told the aristocracy of England that they were the keepers of the public conscience and trustees to the wealth and knowledge of the nation to be held in trust for succeeding generations. This theory proposes that there is more wisdom and patriotism in the judgment of the few than in the heads and the hearts of the many. In medicine there are no lords nor Houses of Lords, no monarchs or monasteries, no aristocracy, save that of intellect, no dignitaries nor diplomates, but every man at once a servant and a king.

